# Assessment of perceived environmental quality in urban regeneration: integrating neurophysiological and psychological perspectives

**DOI:** 10.3389/fpsyg.2026.1874589

**Published:** 2026-07-30

**Authors:** Xin Cheng, Xin Wang, Peisi Xu, Jiaxun Ge, Junyao Ma

**Affiliations:** Department of Landscape Architecture, Xihua University, Chengdu, Sichuan, China

**Keywords:** electroencephalography (EEG), evidence-based management, eye-tracking, perceived environmental quality, urban regeneration

## Abstract

As urban development shifts toward quality-oriented regeneration, evaluating human–environment interactions is critical for sustainable urban management. This study develops a comprehensive physical–physiological–psychological framework to assess micro-urban regeneration in high-density residential contexts. Through the integration with eye-tracking and questionnaire-based evaluations, a highly significant hierarchy of visual salience was identified: Facilities > Vegetation > Buildings > Roads. Although electroencephalography (EEG)-derived measures did not exhibit statistically significant differences across landscape categories, their descriptive numerical variations are reported strictly as exploratory indicators. Correlation analysis reveals visual attention corresponds to positive appraisal only for facilities, while prolonged fixation at aging buildings is associated with a negative trend in subjective satisfaction. Furthermore, the integration of neurophysiological and psychometric data reveals the restorative capacity of vegetation and the high cognitive load associated with degraded built interfaces, while the non-significant neurophysiological results offer critical methodological insights into the complexities of mobile EEG application within micro-urban renewal contexts. The study concludes that sustainable environmental management in urban renewal should prioritize high-quality facilities and the strategic implementation of scattered, high-frequency micro-greenery. Ultimately, this multimodal approach provides an evidence-based paradigm for urban designers and managers to better balance aesthetic quality and neurophysiological well-being in the built environment, fostering long-term urban resilience.

## 1 Introduction

As global urbanization transitions from rapid spatial expansion to quality-oriented regeneration (Martí et al., 2019; Said and Dindar, 2024), existing residential areas in high-density cities are frequently affected by urban infrastructure degradation, inefficient land use, environmental deterioration, and the loss of urban character (Zhang S. et al., 2025). Such degradation significantly impacts residents’ physiological stress and psychological fatigue in their life experience (Benita and Tunçer, 2019; Xiang et al., 2021). For example, Liu and Liao (2025) revealed that the degradation of built environments reduces aesthetic quality and the amount of outdoor contact with surrounding landscapes in space can affect residents’ physiological stress and psychological fatigue. To cope with challenges, urban regeneration has been adopted as an advanced strategy. Urban regeneration, or urban renewal, can be defined as the planned process of reconstructing the urban environment and resource reuse for resolving interrelated spatial, technical, and socio-economic problems, which leads to a smooth transition from the expansion of development land reuse to the comprehensive quality transformation of urban space and lifestyles’ reuse (Wang et al., 2017; Awad and Jung, 2022). Urban regeneration has become one of the key components of urban planning in many countries and regions (Zhao et al., 2024). Wang et al. (2025b) found that the design interventions significantly shift attention distribution and cognitive activation and are beneficial in shaping environmental preference of young adults in urban design for neighborhood regeneration.

Since urban renewal strategy is essentially applied through restructured physical environments, the assessment of perceived environmental quality is naturally linked with its perceived constitutive (or landscape) elements of the physical environment (Ma et al., 2021; Yuan et al., 2024). In the context of environmental perception, the evaluation of urban space is heavily controlled by constitutive general attributes of urban spaces, such as buildings, urban greens, roads, and urban facilities (Li et al., 2020; Chen et al., 2022; Khaledi et al., 2022; Fang et al., 2024; Liu et al., 2025a). Among them, urban greens are widely known for the strong influence on human emotional effects and aesthetic preference (Zhou Y. et al., 2026). For example, Cheng et al. (2026) found that green exposure produces lasting restorative effects through visual conditions, noise levels, and microclimate, and that targeted environmental control can effectively enhance restorativeness in urban settings. Elsadek et al. (2019) found that green facade significantly reduces the activity of the autonomic nervous system and enhances physiological relaxation. Similarly, Kerimova et al. (2022) revealed that green spaces in shared residential courtyards are the key visual anchors which actively mitigate feelings of insecurity. Visual interaction with vegetation has been shown to decrease cognitive load and eye fatigue (Grassini et al., 2019; Lu Y. et al., 2025; Owen et al., 2026). Meanwhile, Chen et al. (2022) observed that individuals’ connection to nature significantly shapes the distribution of visual attention between trees and buildings, with higher relatedness scores positively correlated with longer viewing time directed at buildings relative to vegetation. In addition to natural elements and buildings, public facilities and streets add visual complexity, respectively. Certain physical street elements, such as pavement materials and facilities, highly control spatial attention distribution, quantifying the attentional utility of street scene livability (Wang et al., 2025a).

Traditionally, the assessment of perceived environmental quality has relied heavily on questionnaires or interviews to assess subjective preferences (Daniel, 2001; Siegrist et al., 2008; Wartmann et al., 2021). However, these approaches are prone to recall bias and fail to decode the unconscious mechanism of human-environment interactions (Daniel, 2001). To overcome these methodological challenges, eye-tracking technology has gained popularity as a non-subjective instrument to map visual engagement. Li et al. (2020) demonstrated that fixation duration and fixation counts can be used in urban green space to objectively explain interest, engagement, and comprehension towards particular elements within a landscape scene. In effect, quantification of oculomotor variables effectively characterizes how human attention is influenced by visual features (Dupont et al., 2015, 2016). Although eye-tracking is able to accurately identify what individuals are looking at, it is insufficient to explain the neurophysiological arousal and internal affective changes induced by the environment.

To bridge the gap between visual attention and internal emotional processing, the emerging field of neuroarchitecture has proposed the synthesis of neurophysiological correlates. The review by Lee et al. (2022) illustrated that a high concentration of central nervous system measures, such as electroencephalography (EEG), functional magnetic resonance imaging, electrocardiogram, electrodermal activity, and saliva cortisol, used to assess arousal and stress in built settings, indicates a need for integrating these tools with behavioral and subjective evidence. Wearable biosensors offer objective, real-time metrics for central nervous system arousal indicative of comprehension of human reaction toward spatial configurations (Jabar et al., 2025; Li et al., 2025; Zhao et al., 2025). Modulation of the EEG’s rhythms, such as alpha-band and beta-band variations, can be used as robust biomarkers for quantifying physiological stress, cognitive load, and emotional arousal parameters (Zhang N. et al., 2025; Zhou C. et al., 2026). In addition, multi-sensory environmental stimuli cause significant fluctuations in the EEG rhythms and autonomic measurements, thereby encouraging an internalised attention (Lu X. et al., 2025).

Given the complementary strengths of these technologies, recent environmental research has shown growing interest in multimodal approaches that integrate eye-tracking and neurophysiological sensing. For example, Elsadek et al. (2020) recorded subjects’ neurophysiological and psychological responses through EEG, heart rate variability, skin conductance, and emotion questionnaire, confirming that exposure to green view through a high-rise window, notably enhances mental health and wellbeing, supporting the value of window views to high-rise planning and design. Shemesh et al. (2022) employed mixed EEG and pupillometry measures to quantify affective responses and visual attention to specific architectural geometries. While such integrated methodologies demonstrate considerable potential, the application of these holistic frameworks to investigate the highly complex micro-landscapes of old urban neighborhoods remains limited. In addition, an overlooked limitation in the existing literature is the fragmented collection of multimodal data, which leads to temporal mismatches between physiological arousal and psychological assessments (Liu et al., 2025b).

Despite these significant methodological advancements, spatially explicit assessments of perceived environmental quality in complex urban districts remain rare. To address this critical gap, this study investigates post-renewal landscape perception and preference in Yulin, a typical high-density neighborhood in Chengdu, China. By integrating spatially explicit physical data, eye-tracking, EEG metrics, and subjective surveys, this study seeks to address the following questions: (1) How does specific urban renewal element in old neighborhoods affect visual attention distributions? (2) To what extent do these elements influence unconscious neurophysiological stress? and (3) How do subjective preferences correspond to both neurophysiological and visual patterns? Ultimately, this integrated approach aims to construct a robust physical-physiological-psychological framework, providing urban planners with a scientific, evidence-based paradigm to optimize neighborhood regeneration.

## 2 Methodology

### 2.1 Study area and visual stimuli acquisition

**Figure 1:**
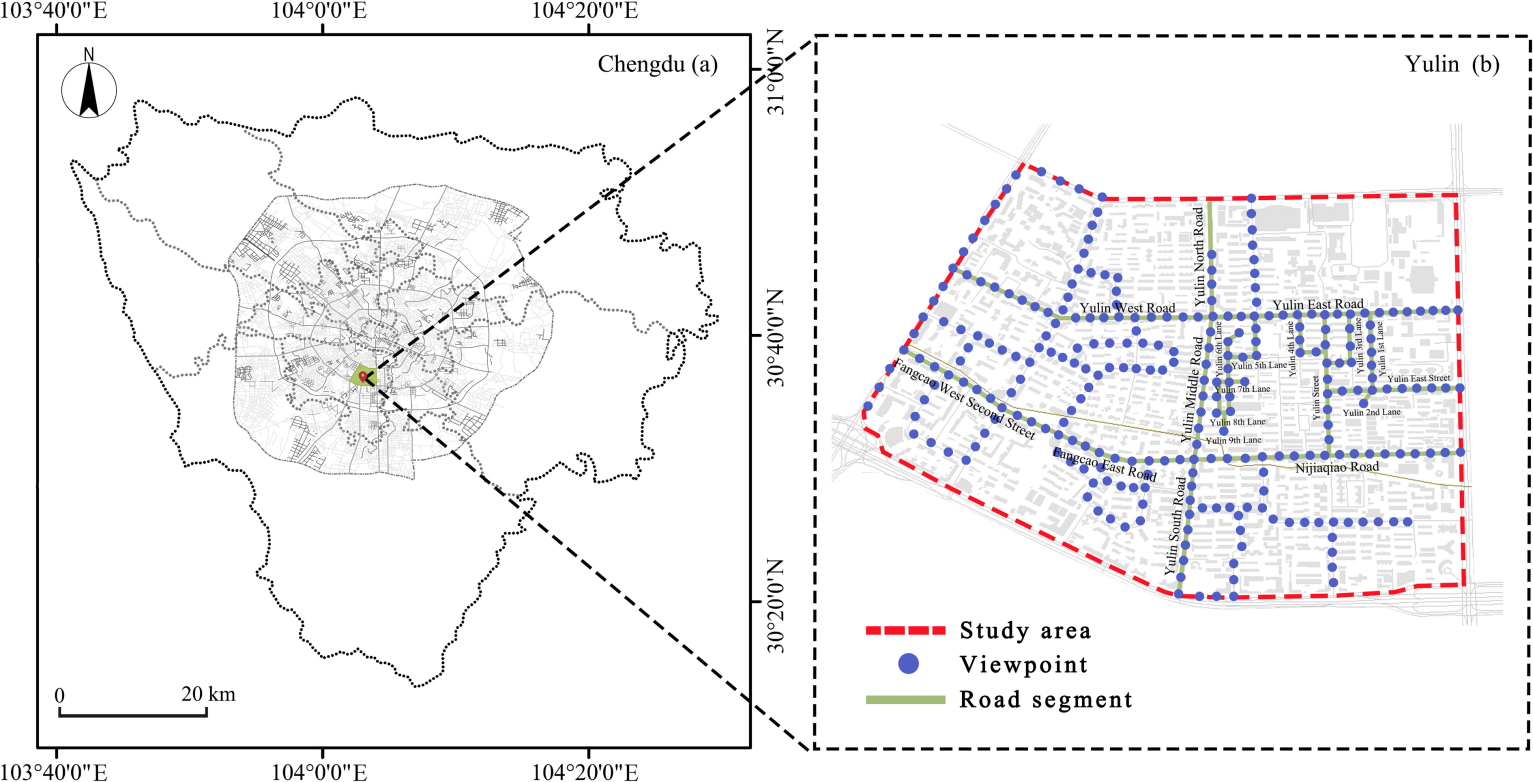
Case study area. **(a)** geographical position of Yulin in Chengdu, China; **(b)** planning map and sampling viewpoints of Yulin.

The empirical research site of this study is the Yulin neighbourhood, Chengdu, Sichuan Province, China (Figure 1). Yulin neighborhood is in the core area of Wuhou District, with a total area of 2.5 km^2^ and a resident population of 73,500. As a representative example of an old district undergoing renewal, Yulin features diverse landscape typologies, including traditional residential clusters, highly commercialized urban streets, and public recreational spaces, making it a representative site for assessing spatially explicit human–environment interactions in the context of urban renewal.

To reduce environmental interference (e.g., noise, temperature) and visual inconsistencies, photographs were used as visual stimuli. Photographs were captured using a DJI Osmo Pocket 3 camera mounted 160 cm from the ground, at an angle equivalent to the average eye height of adult pedestrians. A baseline interval of 25 m between regular street segments was defined and used for spatial continuity. In order to ensure consistency in visual presentation, all photos were taken under clear or uniformly overcast weather conditions, avoiding rain, smog, and strong backlighting. In order to minimize environmental interference from natural light fluctuations, as well as pedestrian and vehicle traffic, image acquisition was conducted during two weekday time windows: morning (08:00–10:00) and afternoon (14:00–17:00), when lighting conditions were stable and street activity was relatively low. This standardized protocol ensured uniform brightness and color temperature across all stimuli, thereby minimizing potential confounding effects on visual attention and subjective questionnaire responses. In total, 273 stimulus images were obtained.

### 2.2 Deep-learning semantic segmentation and spatial quantification

For the objective quantification of physical elements in the streetscape stimuli, the Mask2Former semantic segmentation model was used. Based on the purposes of this research, landscape elements related to urban regeneration were classified into four groups systematically: (1) Vegetation, including all the visible greenery such as street trees, shrubs, potted plants, etc. (2) Buildings, including the major building entities and grey infrastructure, like walls and porches. (3) Roads, including vehicle lanes, pedestrian paths, and paved road surfaces, etc. (4) Facilities, including the public service infrastructure such as street lights, seats, sculptures, signs, and waste bins, etc.

**Figure 2:**
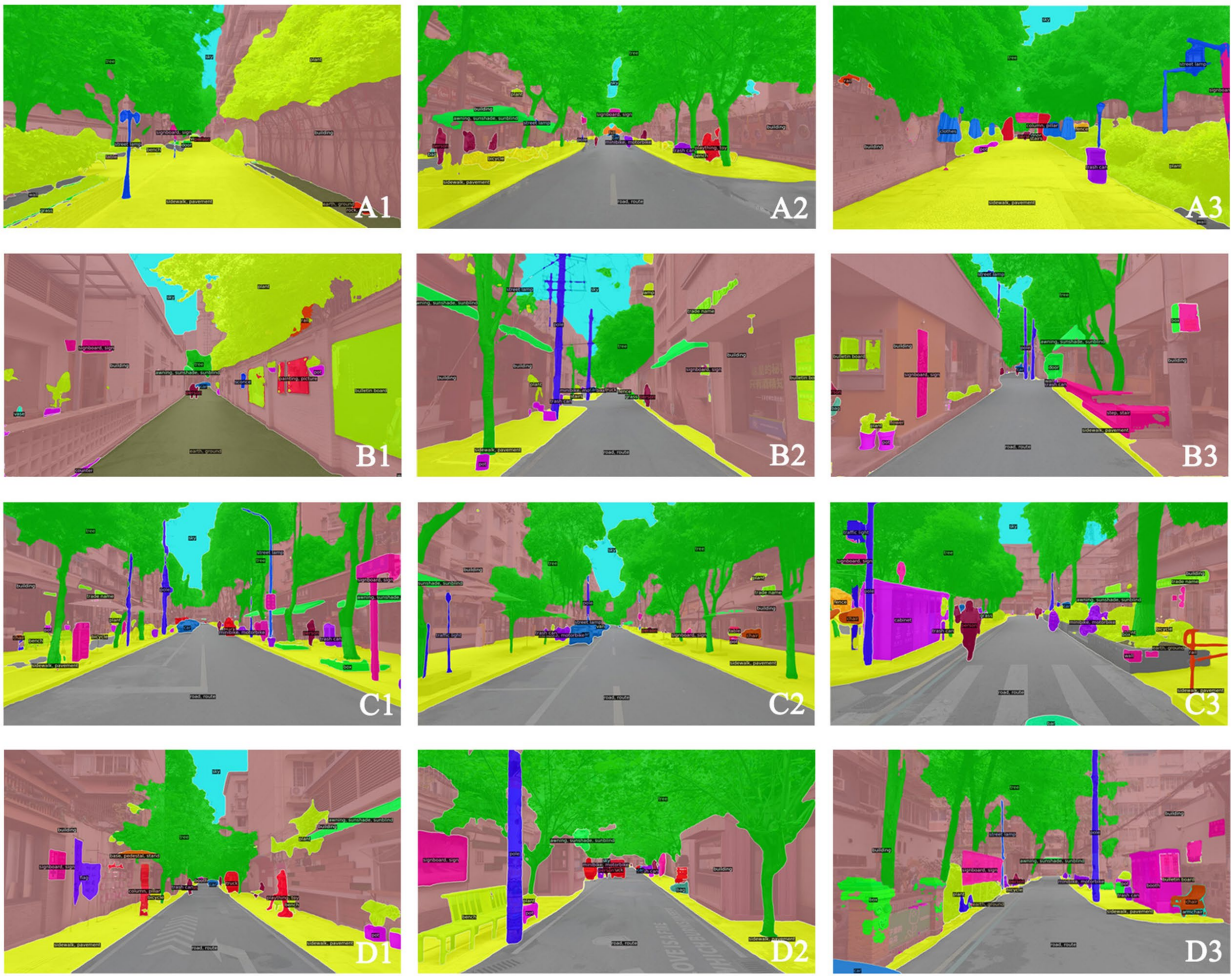
Semantic segmentation results of the experimental stimuli. **(A1−A3)** vegetation, **(B1−B3)** buildings, **(C1−C3)** roads, **(D1−D3)** facilities.

Based on quantitative visual ratios provided by the semantic segmentation, the 273 stimulus images were divided into four groups (A, B, C, D) which could be attributed to vegetation, buildings, roads, and facilities, respectively. To evaluate each landscape element accurately, the dataset was reviewed and evaluated by experts. Specifically, an image was selected if (1) it showed high category purity (the target landscape element was a dominant appearance anchor), and (2) it met contextual coherence with the urban renewal theme. Forty-eight images (12 in each group) were shortlisted after first-round screening. To avoid selection bias, three images were randomly chosen from each group (12 in total) for the final experiment selection (Figure 2, Supplementary file).

### 2.3 Participants and experimental design

Twenty-seven healthy volunteers participated in the experiment. Of the 27 participants (14 male, 13 females; M = 24.7 years old, M = 168 cm in height, Range = 156–183 cm) whose vision is normal/corrected to normal, individual adjustment of the screen height so that it was at each participant’s eye level ensured uniform visual angles while minimizing any issues related to the physical stature of each participant, and a rigorous inclusion/exclusion criteria eliminated any potential confounds resulting from participants having ever lived in the Yulin neighborhood, removing the influences of scene familiarity and long-term memory as confounding factors for physiological baselines. All participants provided written informed consent prior to the experiment (see Supplementary Table 1), and this study was approved by the relevant ethics committee.

**Figure 3:**
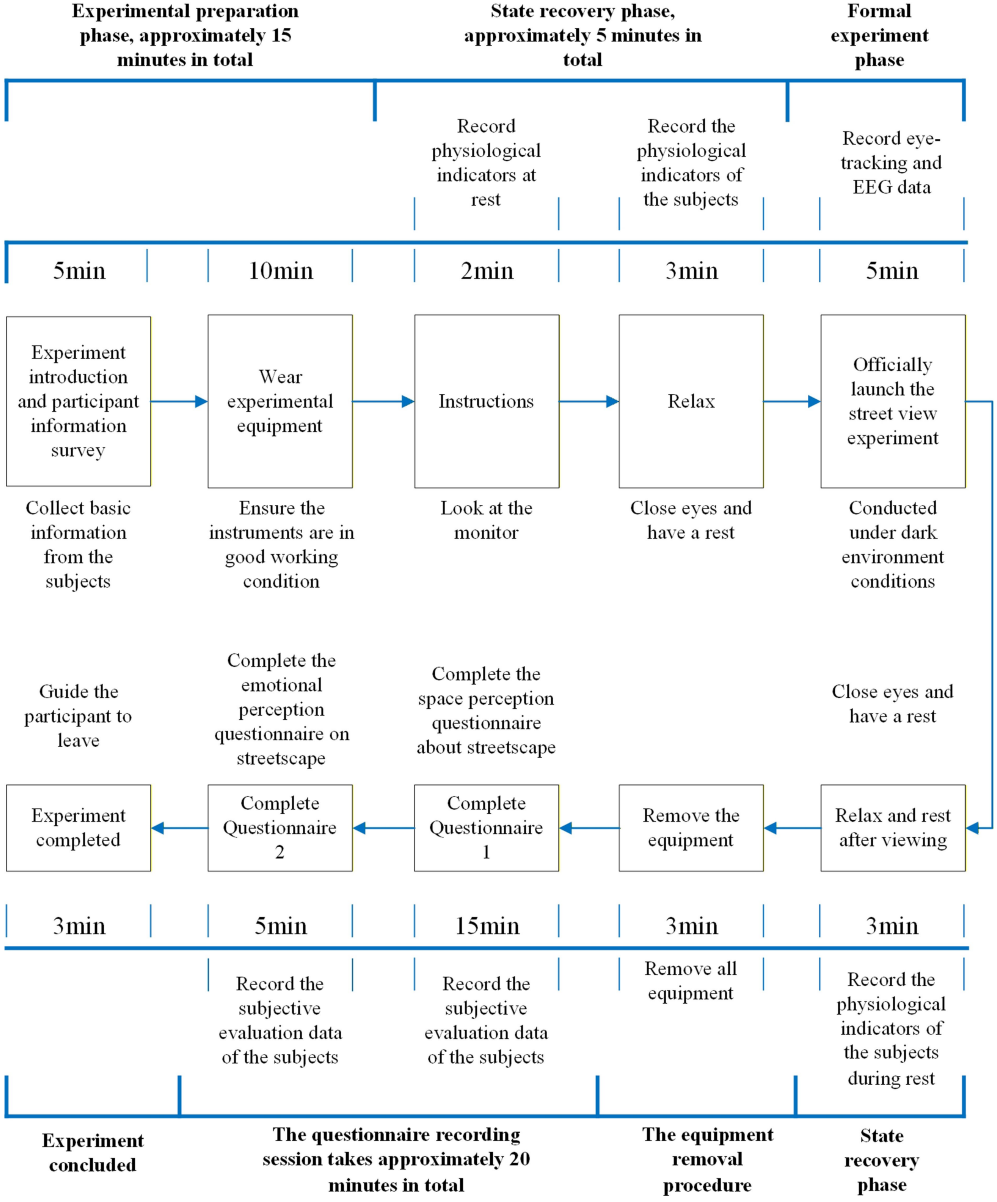
Experimental process.

The experimental protocol was designed to simultaneously collect neurophysiological and oculomotor data with no delays between data collection for reducing the effects of biological responses. Each protocol consisted of a total duration of around 54 min per participant with the protocol structure described below (Figure 3).

1. Preparation and baseline (20 min): device fitting, calibration, and a 5-min resting state recording to establish physiological baselines.
2. Simultaneous multi-modal exposure (5 min): the 12 stimulus images were presented on a high-definition screen in randomized order. Each image was displayed for 20 s, followed immediately by a 5-s blank screen to provide a visual and cognitive rest period. Critically, eye-tracking and EEG data were recorded simultaneously during the 20-s stimulus exposure periods.
3. Recovery phase (6 min): following the stimulus exposure phase, participants were given a resting recovery period, after which all monitoring devices were removed.
4. Psychological assessment (20 min): immediately after physiological data collection, participants completed detailed questionnaires assessing their perceived environmental quality across the four landscape elements and their subjective emotional responses to the urban renewal scenes.

### 2.4 Data processing and analysis

All statistical analyses were performed using IBM SPSS Statistics 27.0, with a significance level of *p* < 0.05 for all tests. Different statistical procedures were applied to oculomotor, neurophysiological, and subjective data to examine the multidimensional datasets.

#### 2.4.1 Eye-tracking data processing and analysis

Eye movements are acquired using the Dikablis Glasses 3 head-mounted binocular eye-tracking system produced by Ergoneers GmbH (Germany). Its binocular infrared tracking sampling rate is 60 Hz and its gaze-tracking spatial accuracy reaches 0.1°–0.3° of visual angle. Its data acquisition and analysis software is D-Lab. With a 1920 × 1,080 full-HD scene camera and providing free-head-movement eye-tracking recordings in natural scenes, four-point pupil calibration, automatic area of interest identification, and multi-dimensional eye-movement indicator statistical export. To analyze the visual attractiveness of the four landscape element groups, four representative eye-tracking indicators were chosen: fixation count (frequency of attention), total fixation duration (TFD; overall attractiveness), mean fixation duration (MFD; information processing depth), and total fixation duration proportion (proportion of attentional allocation weight). A one-way analysis of variance (ANOVA) was performed. Before performing the analysis, Shapiro–Wilk and Levene’s tests revealed that all measures were normally distributed (*p* > 0.05) and had homogeneity of variance (*p* > 0.05). In addition, this study conducted *post hoc* multiple comparisons using the Bonferroni correction for the control of Type I error. Data sets are presented as aggregated box plots (distribution across landscape element grouping) and aggregated heatmaps (by landscape element type) of the 12 experimental stimuli.

#### 2.4.2 Neurophysiological data processing and analysis

EEG data were collected using the Huido Brain Training Analysis System, which employs a NeuroSky portable EEG headset (Bluetooth 4.0) as the front-end acquisition device. The system integrates a TGAM-551 single-channel EEG sensor module, capturing non-invasive signals through a gold-plated dry electrode positioned on the forehead. Operating at a sampling rate of 250 Hz with 12-bit A/D resolution, the device records within an effective frequency bandwidth of 3–125 Hz. Power-line noise is mitigated by a hardware-integrated 50 Hz notch filter. The headset provides spectral power estimates across standard frequency bands (*δ*, *θ*, *α*, *β*, and *γ*), as well as attention and relaxation indices. Based on these outputs, the β/α ratio was used as an indicator of physiological stress, whereas the *θ*/*β* ratio was used as an indicator of cognitive engagement and attentional processing. Repeated-measures ANOVA was used to test subjects’ physiological responses to the four landscape categories, as this method controls for individual differences by removing between-subject variability. Two common physiological measures: β/α, the level of physiological stress, and *θ*/*β*, the level of cognition and attentiveness, were tested to show differences in the physiological response among the landscape categories.

#### 2.4.3 Questionnaire data processing and analysis

Subjective perceived environmental quality was examined by using the two questionnaires, including landscape element characteristics and self-rated perception of emotions (see Supplementary material questionnaires). Reliability was tested using Cronbach’s *α* to verify the data quality. The construct validity was determined by KMO and Bartlett’s test of sphericity in SPSS. A one-way ANOVA was utilized to compare the spatial perception differences and emotional scores among each landscape element group. Based on the model, landscape element was the independent variable, and each aspect evaluation was the dependent variable, respectively. Least Significant Difference (LSD) post-hoc testing was utilized to determine between-group differences.

#### 2.4.4 Multimodal data integration and cross-modal analysis

To overcome the one-dimensional limitations of data and to further investigate the integrated mechanism of urban regeneration perception, the three heterogeneous data sets, namely eye-tracking data, EEG data, and questionnaire data, were jointly integrated in this study for analysis. To eliminate heterogeneity of scale and units, the raw data of all indicators were preprocessed by *Z*-score normalization, which converts each indicator into a normed score with a mean of 0 and a standard deviation of 1. Then, the Pearson correlation coefficients were computed for each landscape element (i.e., buildings, vegetation, roads, and facilities) in order to find pairwise relationships between eye-tracking data (fixation count, TFD, etc.), EEG data (*β*/*α*, *θ*/*β*), and questionnaire data, specifically total Semantic Differential (SD), pleasure, and arousal.

## 3 Results

### 3.1 Visual attention allocation patterns

One-way ANOVA (Table 1) confirmed highly significant differences across all eye-tracking measures (*F* = 23.225–52.204, *p* < 0.001), as different urban renewal elements had different and separate visual attention mechanisms. A clear hierarchy was confirmed by post-hoc Bonferroni analysis (*p* < 0.05) and indicated that facilities were visually predominant and dominated over other types of elements in both fixation count and TFD. Vegetation was the second most visually dominant landscape element with a fixation count of 25 ± 9 and a total fixation duration proportion of 17.35% ± 7.67%, higher than the other two categories. Buildings had a mid-level of visual focus, with a fixation count of 18.17 ± 5.42 and a total fixation duration of 2.94 ± 1.12 s. Whereas roads were the second least attractive across all indicators, with the lowest total fixation duration proportion of 2.28% ± 3.89% and an MFD of 1.21 ± 0.77 s, and significantly different from the other three landscape categories. These findings show that in urban renewal, functional facilities and green infrastructure are the visual anchors, while traditional gray infrastructure elements such as roads have a weak influence on pedestrian visual attention.

**Table 1:** Results of one-way ANOVA of eye-tracking indicators for different urban renewal elements.

Indicators	Fixation count (counts)	Total fixation duration (s)	Mean fixation duration (s)	Total fixation duration proportion (%)
Buildings	18.17 ± 5.42	52.64 ± 25.22	2.94 ± 1.12	11.57 ± 7.08
Vegetation	25 ± 9	77.6 ± 29.83	3.36 ± 1.46	17.35 ± 7.67
Roads	7.65 ± 10.13	11.29 ± 18.95	1.21 ± 0.77	2.28 ± 3.89
Facilities	29.96 ± 10.9	110.81 ± 34.88	3.88 ± 1.16	24.15 ± 7.72
*F*	25.845	52.204	23.225	42.943
*p*	<0.001	<0.001	<0.001	<0.001

MFD is calculated as the group-level mean of trial-level MFD values, where MFD_trial = TFD_trial/Fixation Count_trial for each stimulus image per participant. Therefore, MFD values reported in this table may differ slightly from the ratio of the group-level aggregated TFD mean to the fixation count mean.

**Figure 4:**
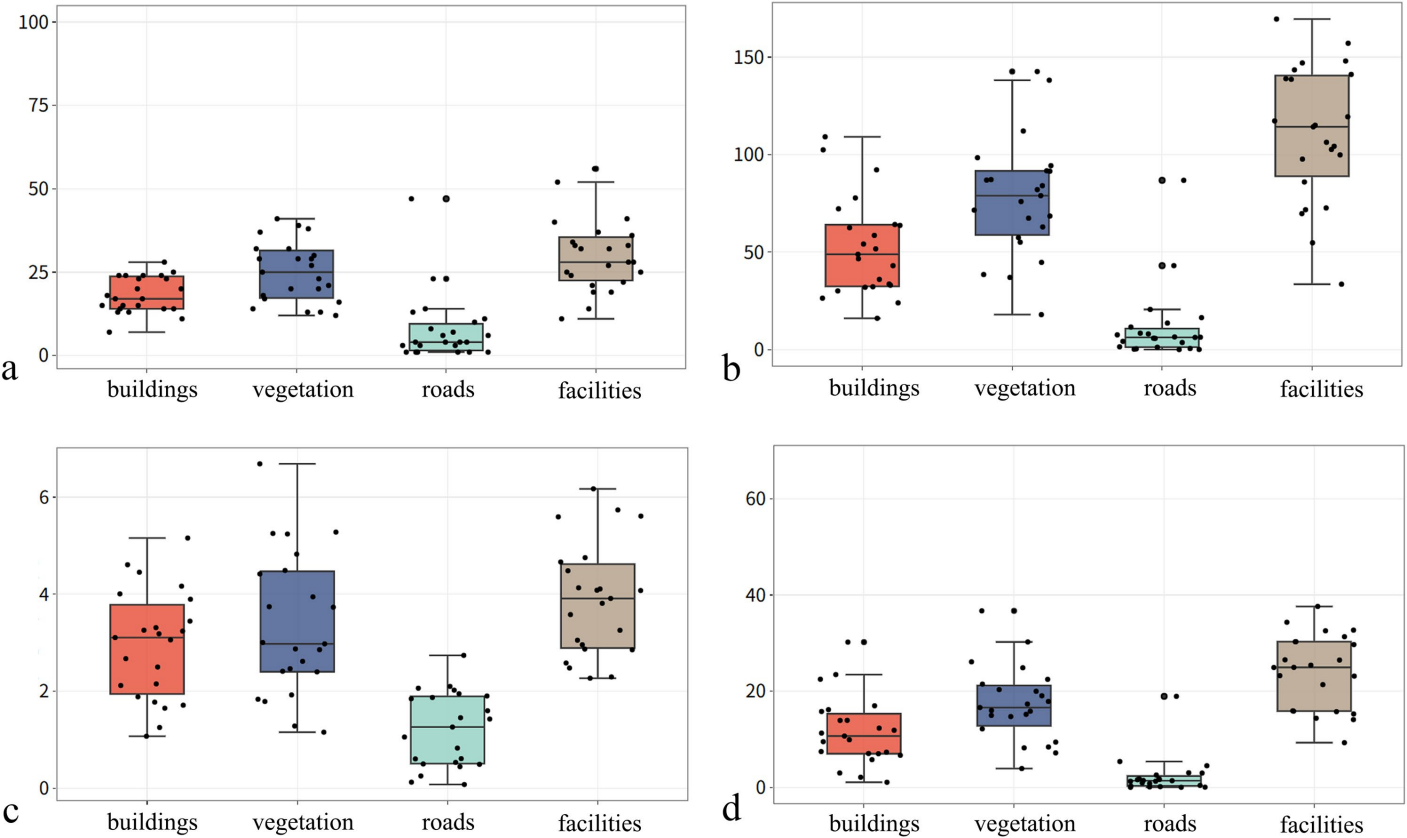
Box plot of four eye-tracking indicators for urban renewal elements. **(a)** fixation count, **(b)** total fixation duration, **(c)** mean fixation duration, **(d)** total fixation duration proportion.

Box plots (Figure 4) also validated the findings, with even hierarchical stratification across all four elements. Facilities remained at the high end of the median values and remained stable within interquartile ranges, proving they continue to excel within attention capture and allocation weight. Vegetation ranks second in both fixation count and TFD, yet its position shifts significantly downward in MFD with higher data dispersion, reflecting individual variability in visual engagement. Buildings occupy a central position overall, with MFD similar to that of facilities but significantly lower fixation count and total fixation duration proportion. Roads rank in the lowest interval across all metrics with no clear anomalies, signifying a uniformly low level of visual interest.

**Figure 5:**
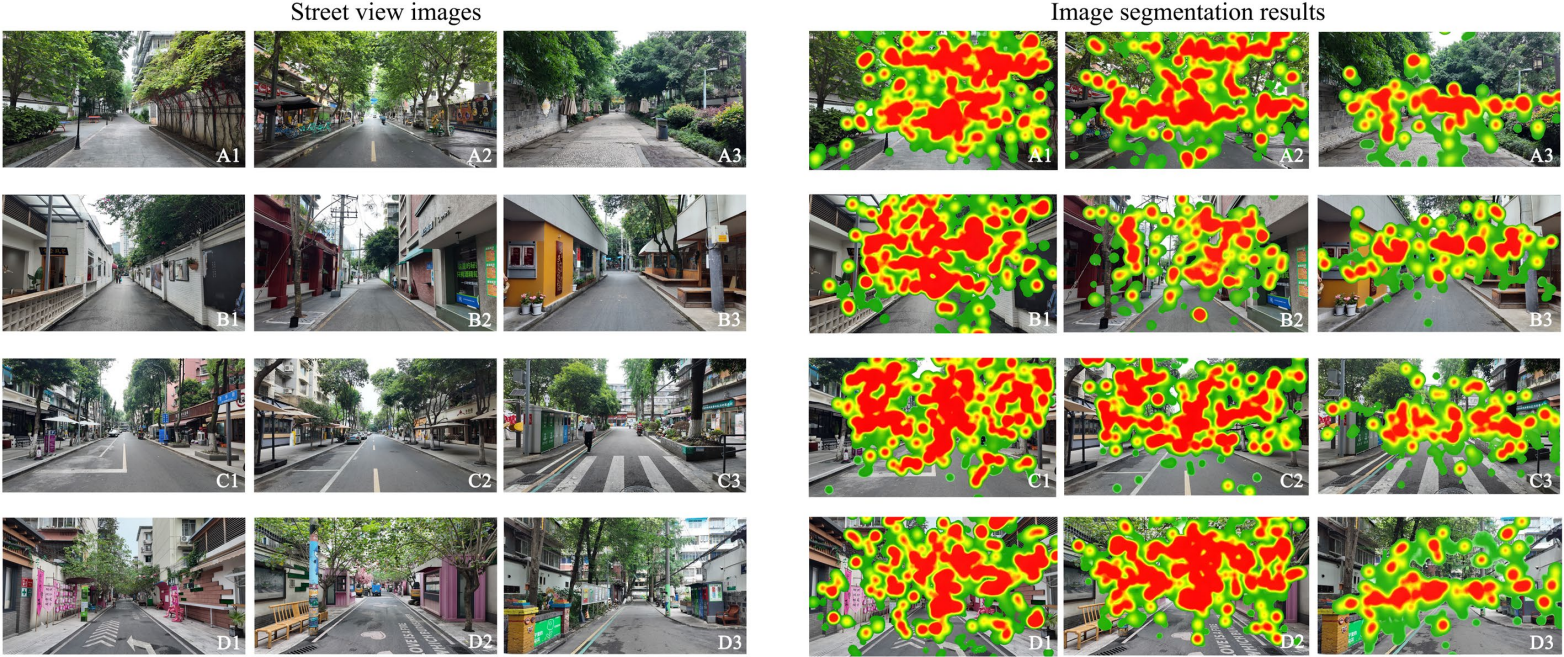
Heat maps of the experimental stimuli. **(A1−A3)** vegetation, **(B1−B3)** buildings, **(C1−C3)** roads, **(D1−D3)** facilities.

For visualizing spatial distribution results, aggregate heatmaps of the 12 streetscape stimuli have been presented in Figure 5 with warmer degrees indicating high visual density and cooler degrees indicating low visual density. All of the core patterns in the heatmaps align well with quantitative findings. Deep red clusters persist around functions of seats and sign-based facilities and buildings as attentional hubs, while vegetation creates diffuse, widely-spreading attention areas hinting at fast attention by vegetal elements. Buildings have limited visual fixations on fine-grained architectural elements, such as decorative elements. Roads and paving remain in blue-to-green low-attention regions without perceptually strong signals around functional nodes, such as crossings. Those heatmaps further justify the visual attraction hierarchy: Facilities > Vegetation > Buildings > Roads.

### 3.2 Neurophysiological response patterns

In this study, a repeated-measures ANOVA was conducted to investigate the effect of the four landscape elements on participants’ EEG metrics (Supplementary Table 2). The data were normally distributed (*p* > 0.05) and met the requirements for parametric testing (Supplementary Table 3). The results showed that the main effect of landscape element was not significant for both the *β*/*α* ratio (*F* = 0.784, *p* = 0.507) and the *θ*/*β* ratio (*F* = 0.597, *p* = 0.619). This finding indicates that, in these experimental conditions, there was no significant effect of the landscape elements on participants’ physiological response in terms of their EEG. The estimated marginal means (Supplementary Figure 1) represent purely descriptive, non-significant numerical fluctuations and must not be interpreted as evidence of a real effect. Consequently, these metrics cannot substantiate any regulatory or inductive effect of specific urban renewal elements on individuals’ underlying physiological states.

### 3.3 Psychometric evaluation of perceived environmental quality

The psychometric survey had high internal consistency and structure. The overall Cronbach *α* was 0.818, indicating strong data reliability. Validity assessment reported an overall KMO (0.804) and significant Bartlett sphericity test (*p* < 0.001). This suggested that the data could be processed for further statistical analysis. One-way ANOVA was performed and revealed significant differences in most perceptual dimensions (SD total, sub-dimensions) and emotional evaluation categories (*p* < 0.05) except for Safety (Q5). Importantly, the overall perception (*F* = 8.722, *p* < 0.001), pleasure (*F* = 9.419, *p* < 0.001), and arousal (*F* = 9.994, *p* < 0.001) were significantly different. This strongly supported that the renewed landscape elements are the dominant factors in user perception and emotional experience. *Post-hoc* LSD tests (*p* < 0.05) further clarified the specific differences between landscape elements (Table 2).

**Table 2:** Evaluation of landscape element characteristics and self-reported emotional ratings under different landscape elements (M ± SD).

Evaluation dimensions	Buildings (M ± SD)	Roads (M ± SD)	Facilities (M ± SD)	Vegetation (M ± SD)	*F*	*p*
SD score	3.51 ± 7.95b	7.49 ± 7.64ab	9.04 ± 7.86a	9.59 ± 7.23a	8.722	<0.001
Q1 vegetation	−0.13 ± 1.35c	0.54 ± 1.35bc	0.48 ± 1.26b	2.38 ± 0.91a	63.281	<0.001
Q2 facilities	−0.67 ± 1.13c	0.78 ± 1.25b	1.03 ± 1.28a	0.12 ± 1.61b	22.566	<0.001
Q3 shading	−0.22 ± 1.49c	−0.07 ± 1.33c	0.54 ± 1.22b	2.03 ± 1.06a	44.017	<0.001
Q4 commercial activity	−0.72 ± 1.33b	1.19 ± 1.19a	−0.77 ± 1.55b	−0.83 ± 2.10b	26.571	<0.001
Q5 safety	0.81 ± 1.39a	1.38 ± 1.00a	1.01 ± 1.35a	1.25 ± 1.36a	2.633	<0.050
Q6 spaciousness	−0.10 ± 1.32c	1.26 ± 1.26a	0.41 ± 1.18b	1.00 ± 1.33ab	15.911	<0.001
Q7 distinctiveness	1.09 ± 1.39ab	0.32 ± 1.54b	1.62 ± 1.27a	0.71 ± 1.36ab	10.964	<0.001
Q8 aesthetics	1.04 ± 1.30ab	0.78 ± 1.04b	1.65 ± 1.27a	1.09 ± 1.07ab	6.696	<0.001
Q9 cleanliness	1.65 ± 1.10a	1.07 ± 1.28b	1.48 ± 1.47ab	1.29 ± 1.21ab	2.650	<0.049
Q10 vividness	0.80 ± 1.09b	0.25 ± 1.12b	1.59 ± 1.14a	0.80 ± 1.25b	17.076	<0.001
Emotion—pleasure	5.67 ± 1.53ab	4.97 ± 1.42b	6.32 ± 1.81a	6.03 ± 1.51a	9.419	<0.001
Emotion—arousal	4.86 ± 1.68ab	4.04 ± 1.62b	5.68 ± 2.06a	4.84 ± 1.62ab	9.994	<0.001

Values in the same row followed by different lowercase letters indicate significant differences at the *p* < 0.05 level.

Regarding landscape characteristic features, total SD scores for vegetation (SDs: 9.59 ± 7.23) and facilities (SDs: 9.04 ± 7.86) were higher than those in buildings (SDs: 3.51 ± 7.95). The total vegetation score was highest in Q1 (vegetation) and Q3 (shading), in line with the function of vegetation as a natural element. Roads scored highest in Q4 (commercial activity) and Q6 (spaciousness), depicting their transport function, while facilities were highest in Q2 (facilities), Q7 (distinctiveness), and Q8 (aesthetics). Q5 (safety) did not show any significant distinction in all scenarios. In terms of self-reported emotional valence, facilities and vegetation also scored higher in pleasure than roads, which had the lowest ranking score of 4.97 ± 1.42; and for the arousal category, they scored the highest with 5.68 ± 2.06. These findings indicate that in terms of their level of interaction, an environment with Cross-modal correlation and subjective–objective integration is incomplete if analyzed through single-dimensional metrics alone, thereby highlighting the necessity of combining physiological gaze behavior with retrospective psychological feedback to capture the full spectrum of human-environment dynamics.

The correlation patterns between objective physiological markers and subjective psychometric assessments exhibited significant variability across the four landscape categories, evidencing that human perception is complex (see Table 3). For the Buildings category, no statistically significant correlations were observed (*p* > 0.05), and most correlation coefficients were negative. Specifically, the trend for fixation count and TFD was negative against total SD score. This indicates that subjective appreciation is not improved with visual attention to building envelopes. The visual attention in this category actually displayed a slightly negative relationship with architectural facade features and may even weaken such appreciation, which may represent an imprecise perceptual process. In contrast, for vegetation, fixation count was moderately associated with pleasure (*r* = 0.347) but very weakly with MFD. For EEG indicators, all were poorly associated with subjective dimensions. For roads, fixation count and MFD show weak positive relationships with the total SD score (*r* = 0.404). For the facility category, it was the strongest correlation relationship. Fixation count was related to total SD score (*r* = 0.435, *p* < 0.05) and TFD was highly related to SD score (*r* = 0.586, *p* < 0.01). This indicates that greater visual attention directed toward facilities directly corresponds to higher perceived environmental quality.

**Table 3:** Correlation analysis of multimodal data for landscape elements in urban renewal.

Urban renewal elements	Indicator categories	Indicators	SD score	Emotion—pleasure	Emotion—arousal
Buildings	Eye-tracking indicators	Fixation count	−0.355	0.066	0.075
		Total fixation duration	−0.311	−0.206	−0.021
		Mean fixation duration	−0.055	−0.311	−0.104
		Total fixation duration proportion	−0.335	−0.205	0.014
	EEG indicators	*β*/*α* ratio	−0.374	−0.346	−0.281
		*θ*/*β* ratio	−0.205	−0.153	−0.282
Vegetation	Eye-tracking indicators	Fixation count	0.314	0.347	0.174
		Total fixation duration	0.051	0.157	0.223
		Mean fixation duration	−0.214	−0.191	0.066
		Total fixation duration proportion	0.143	0.202	0.314
	EEG indicators	*β*/*α* ratio	0.110	−0.055	−0.148
		*θ*/*β* ratio	−0.143	−0.009	−0.202
Roads	Eye-tracking indicators	Fixation count	0.404	0.38	0.153
		Total fixation duration	0.3	0.326	0.086
		Mean fixation duration	0.007	0.073	0.104
		Total fixation duration proportion	0.378	0.338	0.12
	EEG indicators	*β*/*α* ratio	−0.011	−0.127	−0.075
		*θ*/*β* ratio	0.396	0.234	0.137
Facilities	Eye-tracking indicators	Fixation count	0.435*	0.35	0.192
		Total fixation duration	0.639**	0.318	0.255
		Mean fixation duration	0.269	0.056	0.092
		Total fixation duration proportion	0.586**	0.288	0.224
	EEG indicators	*β*/*α* ratio	−0.165	−0.287	−0.469
		*θ*/*β* ratio	0.147	−0.047	−0.216

*p < 0.05; **p < 0.01.

## 4 Discussion

### 4.1 Hierarchy of visual attention

Combining one-way ANOVA and multidimensional boxplots with aggregated heatmaps identifies a hierarchical order of visual salience: Facilities > Vegetation > Buildings > Roads. Facilities are the key visual element, given their dual appeal of practical utility and perceptual complexity (Asadi-Shekari et al., 2019; Tamiami Fachrudin et al., 2024). In second place, vegetation is perceived as restorative buffer elements compared to structural components. This is consistent with the findings of Xiang et al. (2021), who found the contrast between the restorative effects of natural elements and the stress caused by dense building view factors. These results could provide a direct quantitative rationalization of landscape regeneration in high-density residential zones. In addition, this street-level hierarchy provides an interesting contrast to vertical-scale perspective studies. For instance, studies by Pilarczyk et al. (2026) in Krakow revealed that residential buildings and facilities were visually prioritized from window height, while natural elements were largely overlooked. The disparity indicates that visual prioritization is highly sensitive to observation height and spatial scale. From the pedestrian scale, facilities are perceived as more direct anchors than surrounding architecture or natural elements. The combination of multi-dimensionality reinforces that urban regeneration needs to acknowledge heterogeneity of perceived experience across different spatial scales and viewpoints to optimize the subjective-objective quality of the built environment.

### 4.2 Neurophysiological responses to urban regeneration

Crucially, since the repeated-measures ANOVA revealed no statistically significant differences in either the *β*/*α* or *θ*/*β* ratios, there are no discernible actual neurophysiological effects from the visual stimuli. The numerical mean increase in the *β*/*α* ratio for buildings and *θ*/*β* rise for vegetation are simply descriptive differences without statistical significance. Instead of corroborating conclusive changes in physiology, these non-significant outcomes are an important methodological implication for future neuroarchitectural research. Either the inability to generate measurable CNS shifts with short-term static photographic stimuli may be indicative of their limitations for such analysis, or there is simply too much interpersonal variance when parsing high-density urban micro-landscapes. Future studies should employ immersive virtual environments or extended dynamic exposures to help determine whether there are more subtle physiological synchronies at work that go beyond these descriptive numerical variations.

### 4.3 Subjective psychological responses to urban regeneration

The results of the questionnaire give psychosocial validation of the interventions. Vegetation contributed considerably to the maximum Pleasure scores, and this supports the argument raised by Li and Sullivan (2016) on the intrinsic relationship between green exposure and emotional restoration. This reinforces the role of nature-based solutions as a fundamental pillar of perceived environmental quality in dense urban quarters. Facilities received the maximum scores for Distinctiveness, Aesthetics, and Vividness, and the maximum Arousal scores, suggesting that the regeneration of Yulin District have succeeded in providing high-quality facilities to stimulate street vitality. These results demonstrate that the strategic blending of vegetation and distinctive facilities has helped the streetscape become more than a mere traffic corridor, rendering it a vibrant high-quality public space. Counterintuitively, subjective safety scores (Q5) did not vary significantly between the four landscape categories (*p* > 0.05), in line with that particular socio-spatial setting’s neighbourhood profile. After decades of institutionalised urban community grid control, and further reinforced by micro-regeneration efforts (e.g., improved street lighting systems, community-based policing patrols), the perceived homogenous baseline in terms of overall civic security is very much supported throughout the entirety of Yulin. Thus, localized physical degradations (such as aging building facades) or natural variations (vegetation density) do not significantly compromise residents’ fundamental sense of safety, which remains robustly supported by top-down environmental management.

### 4.4 Interpreting the multimodal perception mechanism

Eye-tracking and subjective questionnaire data, supplemented by exploratory descriptive trends from EEG clarify the nonlinear relation between the subject-based and objective indicators of the elements of the urban renewal landscape. The correlations between eye-tracking and subjective evaluations were validated for facilities, while other categories were weak or showed an inconsistent pattern. The reason for the lack of a significant correlation between EEG and the cognitive judgements might be attributed to temporal desynchronization, while local neural oscillations are not synchronized with retrospective global summation. This suggests that under a static and tightly controlled experiment, the stimuli may not provide sufficient automatic arousal for lowering variability among subjects.

Furthermore, A critical divergence was exposed in the perception of vegetation versus buildings. As vegetation is most influenced by discrete and frequent visual stimuli instead of staring, this indicates that longer visual attention to intensive greenery in high-density areas is ideal for restorative benefits. Comparatively, the negative trend observed for buildings indicates that increased visual attention to uniform or visuo-intensely textured building structures could negatively lower the overall perceived value. Fundamentally, this multimodal integration demonstrated that more visual attention time does not necessarily lead to a more positive experience. This research provides an evidence-based foundation for nuanced urban renewal that moves beyond siloed indicators to highlight the need for balancing visual attention, subjective aesthetic values, and potential neurophysiological recovery trends that warrant further field verification.

### 4.5 Practical implications for sustainable urban regeneration

The integrated findings from the Yulin District suggest that sustainable urban regeneration should move beyond superficial aesthetics to consider spatial cognitive efficiency and potential neurophysiological impacts, even though the latter require more robust field validation due to high individual baseline variability. The significant attention–appraisal coupling observed for facilities, which exhibits strong dominance in distinctiveness and vividness characteristics, supports the application of acupuncture in street-level regeneration. Urban acupuncture, as defined by Lerner (2014), refers to small-scale, point-specific micro-interventions designed to catalyze large-scale socio-spatial revitalization across a neighborhood. Vegetation intervention has also proved to have strong restorative capacities and closely linked to shading and pleasure. In space-limited (aging) districts, renewal strategies should no longer focus on large green patches, but turn to the fragmentation of high-frequency green elements that can dissipate high cognitive loads of dense and congested built environments without resorting to large park interventions. The reverse relationship between architectural attention and subjective appraisal (*r* = −0.311) points to a need for multidimensionality in design criteria so as to dissipate the critical gazing effect evoked when users encounter the facial surface of buildings, as excessive visual clutter would weaken overall satisfaction. By integrating convergences and divergences between subjective and objective data, this research brings about a scientific foundation for design science to ensure that future micro-regeneration efforts optimize spatial cognitive distribution while laying the groundwork for exploring long-term neurophysiological restorative benefits.

### 4.6 Limitations and future directions

While this study provides a novel framework for assessing urban interventions, several limitations remain to be addressed in future studies. First, while a controlled laboratory design ensures the operational convenience offered by the paradigm, relying on static visual stimuli, as is typical of most eye-tracking experiments, cannot fully model the multisensory and dynamic nature of real-world streets. Second, the stimulus set used consisted of only 12 photos (3 per category), which, despite rigorous selection and balancing procedures, may not accurately represent the richness of streetscape composition variations throughout the study area. Future work should utilize larger and more varied sets of images in order to enhance representativeness and generalizability. Third, the EEG results did not reach statistical significance, as is common in neurophysiological studies among specific participant groups. This may partially reflect the relatively small sample size (*N* = 27) and possibly insufficient statistical power to detect nuanced neurophysiological differentiation due to the high variance between individuals that has been shown frequently in studies examining EEG measurements. The EEG data were acquired using a NeuroSky-based system, which provides device-derived indices rather than raw EEG signals and may limit sensitivity to subtle effects. Thus, nonsignificant EEG effects cannot be taken as proof of a null result. Future studies can better emulate ecological validity by increasing the experimental sample size and considering mobile EEG for in-situ experiments. Additionally, future studies could explore the long-term psychological impacts of different regeneration stages to provide a more comprehensive temporal dimension for sustainable urban management.

## 5 Conclusion

This study contributes to the urban regeneration literature in both theoretical and practical dimensions. Theoretically, this research builds upon a physical–physiological–psychological evaluation methodology that encompasses objective visual attention, explorative neurophysiological reactions, and subjective appraisal to establish a broader picture of human–environment interaction dynamics within the context of micro-regeneration contexts. Though the primary effects of the EEG metrics (*β*/*α* and *θ*/*β*) were non-significant under controlled laboratory settings, the prominent visual hierarchy and its strong alignment with subjective pleasure and arousal provide clear evidence-based pathways for design interventions.

Practically, the findings indicate that small-scale design interventions can produce significantly varying perceptual responses. Facilities appeared to be the highest-ranked visual attractors and positively linked with the attributes of perceived vibrancy and uniqueness, whereas vegetation had the largest influence on emotional restoration and environmental quality. The findings argue for the use of strategic urban acupuncture techniques and the inclusion of high-frequency green elements in high-density neighborhoods. By providing empirical evidence about the way certain landscape elements affect human perception, this study offers a replicable evaluation framework for evidence-based and human-centered urban regeneration.

## Data Availability

The original contributions presented in the study are included in the article/Supplementary material, further inquiries can be directed to the corresponding author.
