# Supplementary material for: Assessment of perceived environmental quality in urban regeneration: integrating neurophysiological and psychological perspectives
Source: Front Psychol. 2026 Jul 30;17:1874589. doi: 10.3389/fpsyg.2026.1874589 (PMC13467985; doi:10.3389/fpsyg.2026.1874589)
Supplement: Supplementary file 1 [file Supplementary_file_1.docx]

Supplementary Material

Supplementary Table 1. Informed Consent Form

| **Study Title** | Perception of Urban Renewal in Old Urban Districts of Chengdu | | |
| --- | --- | --- | --- |
| **Researchers** | XXX | | |
| **Participant Criteria** | 1. Students or members of the general public, male or female.  2. Normal vision or corrected-to-normal vision with contact lenses (glasses are not allowed).  3. Aged 18–60 years. | | |
| **Contact Address** | XXX | | |
| **Phone Number** | XXX | **Email** | XXX |
| **Purpose of the Study:**  You are voluntarily invited to participate in a study on the perception of landscapes elements in old urban districts of Chengdu. The research aims to understand how people perceive urban renewal through physiological and cognitive responses. | | | |
| **Procedure:**  1. Wear non-invasive eye tracking and EEG equipment.  2. View a series of photographs of streetscapes while your eye movements and brain activity are recorded.  3. Remove the equipment after the session.  4. Complete two brief questionnaires regarding your perceptions. Duration: Approximately 54 minutes. | | | |
| **Cost:**  There is no cost to you for participating in this study. | | | |
| **Potential Risks and Side Effects:**  The procedures involve non-invasive measurement of eye movements and brain electrical activity. Although risks are minimal, some participants may experience temporary discomfort, such as eye strain, mild fatigue, or minor skin irritation from electrodes. There are no known long-term risks associated with these procedures. | | | |
| **Benefits:**  Although participating does not provide direct personal benefits, the study contributes to knowledge in human perception and urban renewal design. | | | |
| **Privacy:**  Results may be published in academic journals, books, or used for teaching purposes. Your name or any identifiable information will not appear in any materials without your consent. | | | |
| **Statement on Injury:**  The procedures are non-invasive and risk of injury is very low. If you experience discomfort or injury directly related to the study, please contact the principal investigator. Necessary medical care will be provided, but no additional compensation will be offered. | | | |
| **Right to Withdraw:** Participation is entirely voluntary. You may withdraw at any time without penalty or loss of benefits. | | | |
| **Investigator Statement:** I have explained the purpose, procedures, potential risks, benefits, and participant rights, and answered all questions to the best of my ability.  **Signature: Date:** | | | |
| **Participant Statement:**  I have been informed about the purpose, procedures, potential risks and benefits of the study. My questions have been answered satisfactorily. I have read this consent form and agree to participate.    **Signature: Date:** | | | |

**Supplementary Questionnaires**

This appendix includes two questionnaires: (1) Evaluation of Landscape Element Characteristics in Urban Renewal in Yulin District and (2) Self-Reported Emotional Ratings in Yulin District. Prior to completing the main questionnaires, participants will first complete the demographic background items, which are designed to collect basic demographic information for academic purposes. All data will be kept strictly confidential and used solely for the purposes of this research project.

1. Gender

- □ Male □ Female

2. Age

- □ < 20 □ 20–30 □ 31–40 □ 41–50 □ 51–60 □ > 60

3. Educational Attainment

- □ Below High School □ High School □ Vocational/Technical School □ Undergraduate □ Postgraduate or above

4. Occupation

- □ Employee □ Retired □ Homemaker □ Student □ Unemployed □ Other

5. Origin

- □ Chengdu Central Districts □ Other Districts/Counties in Chengdu □ Other regions in China

6. Is your academic major Landscape Architecture?

- □ Yes □ No

7. Frequency of visits to the Yulin District:

□ Never visited □ Once a week □ Multiple times a week □ Occasionally

**Evaluation of Landscape Element Characteristics in Urban Renewal in Yulin District**

In this questionnaire, we will conduct a landscape characteristic survey on the street scene photos you using the Semantic Differential (SD) method. There are 10 pairs of positive and negative adjectives relevant to this study. Please mark the characteristics you consider appropriate in the boxes and place a checkmark (√) using the provided 7-point scale. Thank you very much for your cooperation.

Name：　　　　　　　Gender：　　　Age：

**Scene 1：**

| **Number** | **Adjective** | **Completely** | **Very** | **Quite** | **Neutral** | **Quite** | **Very** | **Completely** | **Adjective** |
| --- | --- | --- | --- | --- | --- | --- | --- | --- | --- |
|  |  | **-3** | **-2** | **-1** | **0** | **1** | **2** | **3** |  |
| Q1 | Sparse vegetation |  |  |  |  |  |  |  | Abundant vegetation |
| Q2 | Inadequate facilities |  |  |  |  |  |  |  | Well-equipped facilities |
| Q3 | Weak shade |  |  |  |  |  |  |  | Strong shade |
| Q4 | Limited commercial space |  |  |  |  |  |  |  | Commerciallviable |
| Q5 | Dangerous |  |  |  |  |  |  |  | Safe |
| Q6 | Narrow |  |  |  |  |  |  |  | Spacious |
| Q7 | Unremarkable |  |  |  |  |  |  |  | Distinctive |
| Q8 | Ugly |  |  |  |  |  |  |  | Beautiful |
| Q9 | Cluttered |  |  |  |  |  |  |  | Clean |
| Q10 | Dim |  |  |  |  |  |  |  | Vibrant |

**Scene 2：**

| **Number** | **Adjective** | **Completely** | **Very** | **Quite** | **Neutral** | **Quite** | **Very** | **Completely** | **Adjective** |
| --- | --- | --- | --- | --- | --- | --- | --- | --- | --- |
|  |  | **-3** | **-2** | **-1** | **0** | **1** | **2** | **3** |  |
| Q1 | Sparse vegetation |  |  |  |  |  |  |  | Abundant vegetation |
| Q2 | Inadequate facilities |  |  |  |  |  |  |  | Well-equipped facilities |
| Q3 | Weak shade |  |  |  |  |  |  |  | Strong shade |
| Q4 | Limited commercial space |  |  |  |  |  |  |  | Commerciallviable |
| Q5 | Dangerous |  |  |  |  |  |  |  | Safe |
| Q6 | Narrow |  |  |  |  |  |  |  | Spacious |
| Q7 | Unremarkable |  |  |  |  |  |  |  | Distinctive |
| Q8 | Ugly |  |  |  |  |  |  |  | Beautiful |
| Q9 | Cluttered |  |  |  |  |  |  |  | Clean |
| Q10 | Dim |  |  |  |  |  |  |  | Vibrant |

**Scene 3：**

| **Number** | **Adjective** | **Completely** | **Very** | **Quite** | **Neutral** | **Quite** | **Very** | **Completely** | **Adjective** |
| --- | --- | --- | --- | --- | --- | --- | --- | --- | --- |
|  |  | **-3** | **-2** | **-1** | **0** | **1** | **2** | **3** |  |
| Q1 | Sparse vegetation |  |  |  |  |  |  |  | Abundant vegetation |
| Q2 | Inadequate facilities |  |  |  |  |  |  |  | Well-equipped facilities |
| Q3 | Weak shade |  |  |  |  |  |  |  | Strong shade |
| Q4 | Limited commercial space |  |  |  |  |  |  |  | Commerciallviable |
| Q5 | Dangerous |  |  |  |  |  |  |  | Safe |
| Q6 | Narrow |  |  |  |  |  |  |  | Spacious |
| Q7 | Unremarkable |  |  |  |  |  |  |  | Distinctive |
| Q8 | Ugly |  |  |  |  |  |  |  | Beautiful |
| Q9 | Cluttered |  |  |  |  |  |  |  | Clean |
| Q10 | Dim |  |  |  |  |  |  |  | Vibrant |

**Scene 4：**

| **Number** | **Adjective** | **Completely** | **Very** | **Quite** | **Neutral** | **Quite** | **Very** | **Completely** | **Adjective** |
| --- | --- | --- | --- | --- | --- | --- | --- | --- | --- |
|  |  | **-3** | **-2** | **-1** | **0** | **1** | **2** | **3** |  |
| Q1 | Sparse vegetation |  |  |  |  |  |  |  | Abundant vegetation |
| Q2 | Inadequate facilities |  |  |  |  |  |  |  | Well-equipped facilities |
| Q3 | Weak shade |  |  |  |  |  |  |  | Strong shade |
| Q4 | Limited commercial space |  |  |  |  |  |  |  | Commerciallviable |
| Q5 | Dangerous |  |  |  |  |  |  |  | Safe |
| Q6 | Narrow |  |  |  |  |  |  |  | Spacious |
| Q7 | Unremarkable |  |  |  |  |  |  |  | Distinctive |
| Q8 | Ugly |  |  |  |  |  |  |  | Beautiful |
| Q9 | Cluttered |  |  |  |  |  |  |  | Clean |
| Q10 | Dim |  |  |  |  |  |  |  | Vibrant |

**Scene 5：**

| **Number** | **Adjective** | **Completely** | **Very** | **Quite** | **Neutral** | **Quite** | **Very** | **Completely** | **Adjective** |
| --- | --- | --- | --- | --- | --- | --- | --- | --- | --- |
|  |  | **-3** | **-2** | **-1** | **0** | **1** | **2** | **3** |  |
| Q1 | Sparse vegetation |  |  |  |  |  |  |  | Abundant vegetation |
| Q2 | Inadequate facilities |  |  |  |  |  |  |  | Well-equipped facilities |
| Q3 | Weak shade |  |  |  |  |  |  |  | Strong shade |
| Q4 | Limited commercial space |  |  |  |  |  |  |  | Commerciallviable |
| Q5 | Dangerous |  |  |  |  |  |  |  | Safe |
| Q6 | Narrow |  |  |  |  |  |  |  | Spacious |
| Q7 | Unremarkable |  |  |  |  |  |  |  | Distinctive |
| Q8 | Ugly |  |  |  |  |  |  |  | Beautiful |
| Q9 | Cluttered |  |  |  |  |  |  |  | Clean |
| Q10 | Dim |  |  |  |  |  |  |  | Vibrant |

**Scene 6：**

| **Number** | **Adjective** | **Completely** | **Very** | **Quite** | **Neutral** | **Quite** | **Very** | **Completely** | **Adjective** |
| --- | --- | --- | --- | --- | --- | --- | --- | --- | --- |
|  |  | **-3** | **-2** | **-1** | **0** | **1** | **2** | **3** |  |
| Q1 | Sparse vegetation |  |  |  |  |  |  |  | Abundant vegetation |
| Q2 | Inadequate facilities |  |  |  |  |  |  |  | Well-equipped facilities |
| Q3 | Weak shade |  |  |  |  |  |  |  | Strong shade |
| Q4 | Limited commercial space |  |  |  |  |  |  |  | Commerciallviable |
| Q5 | Dangerous |  |  |  |  |  |  |  | Safe |
| Q6 | Narrow |  |  |  |  |  |  |  | Spacious |
| Q7 | Unremarkable |  |  |  |  |  |  |  | Distinctive |
| Q8 | Ugly |  |  |  |  |  |  |  | Beautiful |
| Q9 | Cluttered |  |  |  |  |  |  |  | Clean |
| Q10 | Dim |  |  |  |  |  |  |  | Vibrant |

**Scene 7：**

| **Number** | **Adjective** | **Completely** | **Very** | **Quite** | **Neutral** | **Quite** | **Very** | **Completely** | **Adjective** |
| --- | --- | --- | --- | --- | --- | --- | --- | --- | --- |
|  |  | **-3** | **-2** | **-1** | **0** | **1** | **2** | **3** |  |
| Q1 | Sparse vegetation |  |  |  |  |  |  |  | Abundant vegetation |
| Q2 | Inadequate facilities |  |  |  |  |  |  |  | Well-equipped facilities |
| Q3 | Weak shade |  |  |  |  |  |  |  | Strong shade |
| Q4 | Limited commercial space |  |  |  |  |  |  |  | Commerciallviable |
| Q5 | Dangerous |  |  |  |  |  |  |  | Safe |
| Q6 | Narrow |  |  |  |  |  |  |  | Spacious |
| Q7 | Unremarkable |  |  |  |  |  |  |  | Distinctive |
| Q8 | Ugly |  |  |  |  |  |  |  | Beautiful |
| Q9 | Cluttered |  |  |  |  |  |  |  | Clean |
| Q10 | Dim |  |  |  |  |  |  |  | Vibrant |

**Scene 8：**

| **Number** | **Adjective** | **Completely** | **Very** | **Quite** | **Neutral** | **Quite** | **Very** | **Completely** | **Adjective** |
| --- | --- | --- | --- | --- | --- | --- | --- | --- | --- |
|  |  | **-3** | **-2** | **-1** | **0** | **1** | **2** | **3** |  |
| Q1 | Sparse vegetation |  |  |  |  |  |  |  | Abundant vegetation |
| Q2 | Inadequate facilities |  |  |  |  |  |  |  | Well-equipped facilities |
| Q3 | Weak shade |  |  |  |  |  |  |  | Strong shade |
| Q4 | Limited commercial space |  |  |  |  |  |  |  | Commerciallviable |
| Q5 | Dangerous |  |  |  |  |  |  |  | Safe |
| Q6 | Narrow |  |  |  |  |  |  |  | Spacious |
| Q7 | Unremarkable |  |  |  |  |  |  |  | Distinctive |
| Q8 | Ugly |  |  |  |  |  |  |  | Beautiful |
| Q9 | Cluttered |  |  |  |  |  |  |  | Clean |
| Q10 | Dim |  |  |  |  |  |  |  | Vibrant |

**Scene 9：**

| **Number** | **Adjective** | **Completely** | **Very** | **Quite** | **Neutral** | **Quite** | **Very** | **Completely** | **Adjective** |
| --- | --- | --- | --- | --- | --- | --- | --- | --- | --- |
|  |  | **-3** | **-2** | **-1** | **0** | **1** | **2** | **3** |  |
| Q1 | Sparse vegetation |  |  |  |  |  |  |  | Abundant vegetation |
| Q2 | Inadequate facilities |  |  |  |  |  |  |  | Well-equipped facilities |
| Q3 | Weak shade |  |  |  |  |  |  |  | Strong shade |
| Q4 | Limited commercial space |  |  |  |  |  |  |  | Commerciallviable |
| Q5 | Dangerous |  |  |  |  |  |  |  | Safe |
| Q6 | Narrow |  |  |  |  |  |  |  | Spacious |
| Q7 | Unremarkable |  |  |  |  |  |  |  | Distinctive |
| Q8 | Ugly |  |  |  |  |  |  |  | Beautiful |
| Q9 | Cluttered |  |  |  |  |  |  |  | Clean |
| Q10 | Dim |  |  |  |  |  |  |  | Vibrant |

**Scene 10：**

| **Number** | **Adjective** | **Completely** | **Very** | **Quite** | **Neutral** | **Quite** | **Very** | **Completely** | **Adjective** |
| --- | --- | --- | --- | --- | --- | --- | --- | --- | --- |
|  |  | **-3** | **-2** | **-1** | **0** | **1** | **2** | **3** |  |
| Q1 | Sparse vegetation |  |  |  |  |  |  |  | Abundant vegetation |
| Q2 | Inadequate facilities |  |  |  |  |  |  |  | Well-equipped facilities |
| Q3 | Weak shade |  |  |  |  |  |  |  | Strong shade |
| Q4 | Limited commercial space |  |  |  |  |  |  |  | Commerciallviable |
| Q5 | Dangerous |  |  |  |  |  |  |  | Safe |
| Q6 | Narrow |  |  |  |  |  |  |  | Spacious |
| Q7 | Unremarkable |  |  |  |  |  |  |  | Distinctive |
| Q8 | Ugly |  |  |  |  |  |  |  | Beautiful |
| Q9 | Cluttered |  |  |  |  |  |  |  | Clean |
| Q10 | Dim |  |  |  |  |  |  |  | Vibrant |

**Scene 11：**

| **Number** | **Adjective** | **Completely** | **Very** | **Quite** | **Neutral** | **Quite** | **Very** | **Completely** | **Adjective** |
| --- | --- | --- | --- | --- | --- | --- | --- | --- | --- |
|  |  | **-3** | **-2** | **-1** | **0** | **1** | **2** | **3** |  |
| Q1 | Sparse vegetation |  |  |  |  |  |  |  | Abundant vegetation |
| Q2 | Inadequate facilities |  |  |  |  |  |  |  | Well-equipped facilities |
| Q3 | Weak shade |  |  |  |  |  |  |  | Strong shade |
| Q4 | Limited commercial space |  |  |  |  |  |  |  | Commerciallviable |
| Q5 | Dangerous |  |  |  |  |  |  |  | Safe |
| Q6 | Narrow |  |  |  |  |  |  |  | Spacious |
| Q7 | Unremarkable |  |  |  |  |  |  |  | Distinctive |
| Q8 | Ugly |  |  |  |  |  |  |  | Beautiful |
| Q9 | Cluttered |  |  |  |  |  |  |  | Clean |
| Q10 | Dim |  |  |  |  |  |  |  | Vibrant |

**Scene 12：**

| **Number** | **Adjective** | **Completely** | **Very** | **Quite** | **Neutral** | **Quite** | **Very** | **Completely** | **Adjective** |
| --- | --- | --- | --- | --- | --- | --- | --- | --- | --- |
|  |  | **-3** | **-2** | **-1** | **0** | **1** | **2** | **3** |  |
| Q1 | Sparse vegetation |  |  |  |  |  |  |  | Abundant vegetation |
| Q2 | Inadequate facilities |  |  |  |  |  |  |  | Well-equipped facilities |
| Q3 | Weak shade |  |  |  |  |  |  |  | Strong shade |
| Q4 | Limited commercial space |  |  |  |  |  |  |  | Commerciallviable |
| Q5 | Dangerous |  |  |  |  |  |  |  | Safe |
| Q6 | Narrow |  |  |  |  |  |  |  | Spacious |
| Q7 | Unremarkable |  |  |  |  |  |  |  | Distinctive |
| Q8 | Ugly |  |  |  |  |  |  |  | Beautiful |
| Q9 | Cluttered |  |  |  |  |  |  |  | Clean |
| Q10 | Dim |  |  |  |  |  |  |  | Vibrant |

**Self-reported emotional ratings in Yulin districts**

**Name:** ________ **Gender:** ________ **Age:** ________

**1. Instructions**

Please read the following descriptions carefully and rate your emotional experience for each of the 12 scenes. Place a checkmark (√) on the number that best reflects your feelings for each dimension.

**2. Evaluation Criteria (9-Point Scale)**

**(1) Valence (Pleasure):** This represents the positive or negative quality of an individual's emotional state. It captures the essence of emotion, whether viewing the scene feels pleasant or unpleasant.

- **Higher scores (approaching 9):** Indicate a more pleasant state (e.g., happiness, joy, or interest).
- **Lower scores (approaching 1):** Indicate a more unpleasant state (e.g., unhappiness, fear, or anger).

**(2) Arousal （excited）:** This refers to the level of neurophysiological activation and alertness, relating to the mobilization of energy associated with an emotional state. It represents whether the scene feels exciting/stimulating or listless/dull.

- **Higher scores (approaching 9):** Indicate higher arousal (e.g., being visually struck, stimulated, excited, or tense).
- **Lower scores (approaching 1):** Indicate lower arousal (e.g., feeling relaxed, calm, listless, or bored).

**Scene Ratings (1–12)**

**Note:** The following table applies to each of the 12 scenes presented in the experiment.

**Scene 1：**

|  | **1** | **2** | **3** | **4** | **5** | **6** | **7** | **8** | **9** |
| --- | --- | --- | --- | --- | --- | --- | --- | --- | --- |
| **Valence (1 = very unpleasant, 9 = very pleasant)** |  |  |  |  |  |  |  |  |  |
| **Arousal (1 = very not excited, 9 = very excited)** |  |  |  |  |  |  |  |  |  |

**Scene 2：**

|  | **1** | **2** | **3** | **4** | **5** | **6** | **7** | **8** | **9** |
| --- | --- | --- | --- | --- | --- | --- | --- | --- | --- |
| **Valence (1 = very unpleasant, 9 = very pleasant)** |  |  |  |  |  |  |  |  |  |
| **Arousal (1 = very not excited, 9 = very excited)** |  |  |  |  |  |  |  |  |  |

**Scene 3：**

|  | **1** | **2** | **3** | **4** | **5** | **6** | **7** | **8** | **9** |
| --- | --- | --- | --- | --- | --- | --- | --- | --- | --- |
| **Valence (1 = very unpleasant, 9 = very pleasant)** |  |  |  |  |  |  |  |  |  |
| **Arousal (1 = very not excited, 9 = very excited)** |  |  |  |  |  |  |  |  |  |

**Scene 4：**

|  | **1** | **2** | **3** | **4** | **5** | **6** | **7** | **8** | **9** |
| --- | --- | --- | --- | --- | --- | --- | --- | --- | --- |
| **Valence (1 = very unpleasant, 9 = very pleasant)** |  |  |  |  |  |  |  |  |  |
| **Arousal (1 = very not excited, 9 = very excited)** |  |  |  |  |  |  |  |  |  |

**Scene 5：**

|  | **1** | **2** | **3** | **4** | **5** | **6** | **7** | **8** | **9** |
| --- | --- | --- | --- | --- | --- | --- | --- | --- | --- |
| **Valence (1 = very unpleasant, 9 = very pleasant)** |  |  |  |  |  |  |  |  |  |
| **Arousal (1 = very not excited, 9 = very excited)** |  |  |  |  |  |  |  |  |  |

**Scene 6：**

|  | **1** | **2** | **3** | **4** | **5** | **6** | **7** | **8** | **9** |
| --- | --- | --- | --- | --- | --- | --- | --- | --- | --- |
| **Valence (1 = very unpleasant, 9 = very pleasant)** |  |  |  |  |  |  |  |  |  |
| **Arousal (1 = very not excited, 9 = very excited)** |  |  |  |  |  |  |  |  |  |

**Scene 7：**

|  | **1** | **2** | **3** | **4** | **5** | **6** | **7** | **8** | **9** |
| --- | --- | --- | --- | --- | --- | --- | --- | --- | --- |
| **Valence (1 = very unpleasant, 9 = very pleasant)** |  |  |  |  |  |  |  |  |  |
| **Arousal (1 = very not excited, 9 = very excited)** |  |  |  |  |  |  |  |  |  |

**Scene 8：**

|  | **1** | **2** | **3** | **4** | **5** | **6** | **7** | **8** | **9** |
| --- | --- | --- | --- | --- | --- | --- | --- | --- | --- |
| **Valence (1 = very unpleasant, 9 = very pleasant)** |  |  |  |  |  |  |  |  |  |
| **Arousal (1 = very not excited, 9 = very excited)** |  |  |  |  |  |  |  |  |  |

**Scene 9:**

|  | **1** | **2** | **3** | **4** | **5** | **6** | **7** | **8** | **9** |
| --- | --- | --- | --- | --- | --- | --- | --- | --- | --- |
| **Valence (1 = very unpleasant, 9 = very pleasant)** |  |  |  |  |  |  |  |  |  |
| **Arousal (1 = very not excited, 9 = very excited)** |  |  |  |  |  |  |  |  |  |

**Scene 10：**

|  | **1** | **2** | **3** | **4** | **5** | **6** | **7** | **8** | **9** |
| --- | --- | --- | --- | --- | --- | --- | --- | --- | --- |
| **Valence (1 = very unpleasant, 9 = very pleasant)** |  |  |  |  |  |  |  |  |  |
| **Arousal (1 = very not excited, 9 = very excited)** |  |  |  |  |  |  |  |  |  |

**Scene 11：**

|  | **1** | **2** | **3** | **4** | **5** | **6** | **7** | **8** | **9** |
| --- | --- | --- | --- | --- | --- | --- | --- | --- | --- |
| **Valence (1 = very unpleasant, 9 = very pleasant)** |  |  |  |  |  |  |  |  |  |
| **Arousal (1 = very not excited, 9 = very excited)** |  |  |  |  |  |  |  |  |  |

**Scene 12：**

|  | **1** | **2** | **3** | **4** | **5** | **6** | **7** | **8** | **9** |
| --- | --- | --- | --- | --- | --- | --- | --- | --- | --- |
| **Valence (1 = very unpleasant, 9 = very pleasant)** |  |  |  |  |  |  |  |  |  |
| **Arousal (1 = very not excited, 9 = very excited)** |  |  |  |  |  |  |  |  |  |

Supplementary Table 2. Shapiro–Wilk normality test results

|  | **Landscape elements** | **Statistical** | **degrees of freedom** | **significance** |
| --- | --- | --- | --- | --- |
| **β/α ratio** | Roads | 0.972 | 69 | 0.12 |
|  | Buildings | 0.99 | 69 | 0.862 |
|  | Facilities | 0.984 | 69 | 0.508 |
|  | Vegetation | 0.987 | 69 | 0.717 |
| **θ/β ratio** | Roads | 0.985 | 69 | 0.575 |
|  | Buildings | 0.98 | 69 | 0.331 |
|  | Facilities | 0.974 | 69 | 0.17 |
|  | Vegetation | 0.982 | 69 | 0.443 |

Supplementary Table 3. Results of the difference test of core EEG indicators under different landscape elements (M±SD)

| **EEG indicators** | **Buildings** | **Roads** | **Facilities** | **Vegetation** | **F** | **P** |
| --- | --- | --- | --- | --- | --- | --- |
| β/α ratio | 1.19±0.09 | 1.17±0.08 | 1.17±0.06 | 1.17±0.09 | 0.784 | 0.507 |
| θ/β ratio | 0.65±0.04 | 0.65±0.06 | 0.66±0.05 | 0.66±0.06 | 0.597 | 0.619 |

Supplementary Figure 1. Marginal means of EEG indicators under different landscape elements. (a) β/α ratio; (b) θ/β ratio
